# Overexpression of *TaNAC2D* Displays Opposite Responses to Abiotic Stresses between Seedling and Mature Stage of Transgenic *Arabidopsis*

**DOI:** 10.3389/fpls.2016.01754

**Published:** 2016-11-23

**Authors:** Quanjun Huang, Yan Wang

**Affiliations:** ^1^Key Laboratory of Genetic Development and Germplasm Enhancement of Rare Plants in Three Gorges Area, College of Biology and Pharmacy, China Three Gorges UniversityYichang, China; ^2^The Genetic Engineering International Cooperation Base of Ministry of Science and Technology, Key Laboratory of Molecular Biophysics of Ministry of Education, College of Life Science and Technology, Huazhong University of Science and TechnologyWuhan, China

**Keywords:** wheat, *Arabidopsis*, NAC, *TaNAC2D*, stress

## Abstract

Environmental stresses frequently affect plant growth and development, and many genes have been found to be induced by unfavorable environmental conditions. Here, we reported the biological functions of *TaNAC2D*, a stress-related *NAC* (NAM, ATAF, and CUC) gene from wheat. TaNAC2D showed transcriptional activator activity in yeast. TaNAC2D-GFP fusion protein was localized in the nucleus of wheat mesophyll protoplasts. *TaNAC2D* transcript abundance was significantly induced by NaCl, PEG6000, and abscisic acid (ABA) at seedling stage, and repressed by NaCl and PEG6000 at mature plant stage. When *TaNAC2D* was introduced into *Arabidopsis*, the 35-day-old soil-grown *TaNAC2D*-overexpression (*TaNAC2D*-OX) plants displayed slower stomatal closure, higher water loss rate, and more sensitivity to salt and drought stresses compared with WT plants. In contrast, *TaNAC2D*-OX seedlings, grown on 1/2 MS medium supplemented with different concentrations of NaCl, Mannitol, and MV, had enhanced tolerances to salt, osmotic and oxidative stresses during seed germination and post-germination periods. The opposite stress-responsive phenotypes of transgenic *Arabidopsis* were consistent with the expression patterns of *TaNAC2D* in wheat. Moreover, under high salinity and dehydration conditions, three marker genes, including *NCED3*, *RD29A*, and *RD29B*, were down-regulated in 35-day-old *TaNAC2D-*OX plants grown in soil and up-regulated in 14-day-old *TaNAC2D-*OX seedlings grown on 1/2 MS medium. Our results suggest that the change in growth stages and environmental conditions may regulate TaNAC2D’s function.

## Introduction

Environmental stresses may adversely affect plant development and yield. To protect plant against environmental stresses, many genes have been induced, and the products of these stress-inducible genes may provide a protection from stress damage. Stress-inducible genes are largely regulated by transcription factors (TFs). Transgenic plants overexpressing TF genes often show enhanced stress tolerance. For example, overexpression of *TaASR1* in *Nicotiana tabacum* displays improved tolerance to dehydration and oxidative stresses (Hu et al., 2013). *Arabidopsis* plants overexpressing *GsWRKY20* result in decreased water loss and increased drought tolerance (Luo et al., 2013). In rice (*Oryza sativa*), overexpression of *OsMYB2* exhibits enhanced stress tolerance and increased sensitivity to exogenous ABA (Yang et al., 2012).

NAC TFs have been reported to participate in various plant developmental processes, including cell division (Kim et al., 2006), senescence (Yang et al., 2011; Wu et al., 2012; Kim et al., 2013), secondary cell wall formation (Zhong et al., 2010), hormone metabolism (Huh et al., 2012), light responses (Peng et al., 2015), and biotic/abiotic stress responses (Delessert et al., 2005; Wu et al., 2009). Genome-wide transcriptome analysis reveals that ∼20–25% *NAC* genes are involved in stress response (Puranik et al., 2012). Expression patterns of *OsNAC* family show that 140 putative *OsNAC* genes were predicted and at least 15% (21/140) *OsNAC* genes were induced by salt or drought (Fang et al., 2008). Recently, a new study has further revealed that ∼42% (63/149) *OsNAC* genes displayed overlapping expression patterns under biotic and abiotic stresses by microarray data analysis (Sun et al., 2015). Systematic sequence analysis of *Brachypodium distachyon* shows that 101 putative *BdNAC* genes were identified, 18 of which participated in stress responses (You et al., 2015). Moreover, to date many NAC TFs are responsive to abiotic stress in several plant species. Transgenic *Arabidopsis* plants overexpressing *ATAF1*/*ANAC002*, *ANAC019*, *ANAC055*, or *ANAC072* have improved drought tolerance and hypersensitivity to exogenous ABA (Fujita et al., 2004; Tran et al., 2004; Wu et al., 2009). Overexpression of *ANAC016* has low drought tolerance, while *anac016* mutant plants exhibit high drought tolerance (Sakuraba et al., 2015). In rice, transgenic plants overexpressing *OsNAC5*, *OsNAC9*, or *OsNAC10* show improved drought tolerance due to increased root diameter (Jeong et al., 2010, 2013; Redillas et al., 2012). *SNAC1*-overexpressing rice plants exhibit enhanced tolerance to abiotic stresses, increased ABA sensitivity, and decreased water loss rate through closing more stomatal pores (Hu et al., 2006). Compared with the model plant *Arabidopsis* and rice, to date only a few NAC TFs have been revealed to participate in the process of stress in bread wheat (*Triticum aestivum*). Plants overexpressing *TaNAC2*, *TaNAC29*, or *TaNAC67* display improved tolerances to multiple abiotic stresses (Mao et al., 2012, 2014; Huang et al., 2015). Transgenic wheat overexpressing *TaNAC69* shows a conspicuous drought-tolerant phenotype (Xue et al., 2011). Recently, a new study reveals that *TaNAC2-5A*-overexpressing wheat plants produce a higher grain yield through acquired more nitrogen from fertilizer, suggesting that *TaNAC2-5A* participates in the wheat nitrate-signaling pathway (He et al., 2015).

In this study, the full-length cDNA of *TaNAC2D* with high sequence identity to *TaNAC2* was isolated from wheat. Gene expression patterns in response to salinity, dehydration, ABA, and oxidative stresses were examined by qRT-PCR in wheat. Functional analyses revealed that the 35-day-old soil-grown *TaNAC2D*-OX plants displayed reduced tolerance to salt and drought stresses, and slower ABA-mediated stomatal closure compared with WT plants. In contrast, *TaNAC2D*-OX seedlings, grown on 1/2 MS medium supplemented with 100 mM NaCl, 200 mM Mannitol, 1 μM MV, or 1 μM ABA, showed increased stress tolerance and reduced sensitivity to exogenous ABA during seed germination and post-germination periods. The opposite stress-responsive phenotypes of *TaNAC2D*-OX plants were caused by the change in growth stages and environmental conditions. Our results indicate that *TaNAC2D* may have a potential application value in genetic engineering of wheat.

## Materials and Methods

### Cloning and Bioinformatic Analysis of *TaNAC2D*

*TaNAC2D* (GenBank: GQ231954.1) was cloned from wheat (*T. aestivum* L. cv. Chinese spring) using gene-specific primers (**Supplementary Table S1A**). The NAC domain was searched using InterProScan tool^1^. Multiple sequence alignments were carried out by MEGA 5.0 software. The phylogenetic trees were generated using maximum likelihood method, and the bootstrap parameter was set at 1000 replicates. The ID regions of TaNAC2D were predicted using the program PONDR VL3^2^.

### Stress Treatments and qRT-PCR Analysis

Wheat seedlings were grown in the growth chamber at 22°C under a 12-h light/12-h dark cycle. The 10-day-old seedlings grown in petri dish with water were, respectively, exposed to different solutions, including 200 mM NaCl, 10 mM H_2_O_2_, 20% PEG6000, and 100 μM ABA. The 50-day-old mature plants grown in soil were uprooted and treated under the same stress conditions above. Mock-treated seedlings were used as control. Leaves were collected after 0, 1, 3, 6, 12, and 24 h post-treatment for gene expression analysis. Various organs of wheat were collected for organ-specific expression analysis. Total RNA were isolated from those collected materials, and then reverse-transcribed into cDNA using PrimeScript RT reagent Kit with gDNA Eraser (Tiangen, Beijing, China). The qRT-PCR analysis was performed using specific primers (**Supplementary Table S1B**) on a real time PCR machine (Bio-rad, Hercules, CA, USA). Each sample was analyzed using three biological replicates each with three technical replicates. The obtained values were calculated using the 2^-ΔΔCT^ method (Livak and Schmittgen, 2001). *TaActin* (GenBank: AB181991.1) was used as an internal control.

### Subcellular Localization of TaNAC2D

For subcellular localization assay, the recombinant construct of pMD18-35S-GFP (control vector) was firstly obtained as previously described (Huang et al., 2015). The *TaNAC2D* ORF, which is lacking the termination codon, was cloned into pMD18-35S-GFP control vector using specific primers (**Supplementary Table S1C**). The recombinant pMD18-35S-TaNAC2D-GFP vector and control vector were transformed into wheat mesophyll protoplasts according to previous described methods (Yoo et al., 2007). The GFP signal was visualized by fluorescence microscopy. The 4′,6-diamidino-2-phenylindole (DAPI) nuclear stain was used to determine the location of nucleus.

### Transactivation Activity Analysis of TaNAC2D

The *TaNAC2D* ORF (TaNAC2D_1-327_) and two truncated versions (TaNAC2D_1-172_, and TaNAC2D_173-327_) were cloned into pGBKT7 vector (Clontech, USA) using specific primers (**Supplementary Table S1D**). Three constructed vectors and the pGBKT7 were separately transformed into AH109 (Clontech). The transactivation activity of TaNAC2D was evaluated based on the growth characteristic of transformants on SD medium (-Trp/-His/-Ade). X-α-Gal was used for the detection of α-galactosidase activity.

### Generation of Transgenic *Arabidopsis* Plants

The *TaNAC2D* ORF containing the termination codon was cloned into the pBI121 using specific primers (**Supplementary Table S1E**). The pBI121-TaNAC2D plasmid and the pBI121 control vector were separately transformed into *Arabidopsis* plants by *Agrobacterium*-mediated floral dipping method (Clough and Bent, 1998). Seeds harvested from transgenic plants were screened on 1/2 MS medium supplemented with 50 mg/L kanamycin. The expression of *TaNAC2D* was detected by semi-quantitative analysis. Homozygous T_3_ progenies were used for further experiments.

### Assessment of Stress Tolerance in *TaNAC2D*-OX Lines

For the analysis of germination and root growth, *Arabidopsis* seeds of WT, EV and *TaNAC2D*-OX lines were sown on 1/2 MS medium supplemented with 100 mM NaCl, 200 mM mannitol, 1 μM MV, and 1 μM ABA. After 3 days of cold treatment, the seeded plates were incubated in the growth chamber at 22°C with 16-h light/8-h dark cycle. Germination (seedlings with expanded cotyledons) and seedlings emergence (seedlings with green cotyledons) were examined 8 days. Root growth and plant survival of vertically grown seedlings were examined 8 or 21 days.

To assess stresses tolerance, WT and *TaNAC2D*-OX lines were grown in soil under non-stress conditions for 35 days. Subsequently, the 35-day-old plants were irrigated with 200 mM NaCl for 30 days after withholding of water for 2 weeks. For the drought tolerance assay, the 35-day-old plants were not irrigated for 20 days, and then re-watered for 7 days.

To further evaluate the function mechanism of T*aNAC2D*-OX lines to drought stress, the rosette leaves of 35-day-old soil-grown plants were sampled for the assays of water loss and stomatal movement. For assessing the degree of water loss, the detached leaves were placed on filter paper, and then dehydrated for 2 or 3 h at 25°C. Leaf shapes were photographed after dehydration treatment. Moreover, the rate of water loss was measured (*n* = 10). The assay of ABA-induced stomatal closure was further performed according to previous described methods (Osakabe et al., 2013; Ha et al., 2014). The leaf epidermal strips of 35-day-old soil-grown lines were soaked in buffer solution (0.2 mM CaCl_2_, 10 mM KCl, and 10 mM Mes-KOH, pH 6.15) for 6 h under light intensity of 300 μmol⋅m^-2^⋅s^-1^. Subsequently, the epidermal strips were exposed to 0 or 30 μM ABA solution for 1 h. The stomatal movement in guard cells was observed by light microcopy. The size of stomatal apertures was measured for WT and *TaNAC2D*-OX plants (*n* = 50).

### Expression Analyses of *NCED3*, *RD29A*, and *RD29B*

To further investigate the molecular mechanism of stress tolerance, the expression levels of marker genes were detected in WT and *TaNAC2D*-OX plants. The 35-day-old soil-grown plants were submerged in 200 mM NaCl solution for 6 h or placed on dry filter paper, and then dehydrated for 3 h. The 14-day-old seedlings, grown on 1/2 MS medium, were incubated in 1/2 MS liquid medium containing 200 mM NaCl for 6 h or dehydrated for 1 h. The total RNAs were extracted from the whole plants. The qRT-PCR was performed using specific primers (**Supplementary Table S1F**) for the expression levels of marker genes, including *NCED3* (nine-cis-epoxycarotenoid dioxygenase 3; *At3g14440*), *RD29A* (responsive-to-desiccation 29A; *At5g52310*), and *RD29B* (responsive-to-desiccation 29B; *At5g52300*). *Actin2* (*At3g18780*) was used as a reference gene.

### Measurement of H_2_O_2_ Content and Relative Electrolytic Leakage

The 35-day-old soil-grown plants were subjected to salt stress (200 mM NaCl) for 10 days or drought stress for 17 days, rosette leaves of WT and *TaNAC2D*-OX plants were collected to determine H_2_O_2_ content and REL. The same measured methods were used as previously described (Hu et al., 2012; Huang et al., 2015).

## Results

### *TaNAC2D* Encodes a Plant-Specific NAC Transcription Factor

*TaNAC2D* (GenBank: GQ231954.1) was cloned from bread wheat. The cDNA sequence of *TaNAC2D* contains a 984 bp ORF that encodes a protein of 327 amino acid residues. Sequence alignment and phylogenetic analyses (**Supplementary Figures S1A** and **S2**) revealed that TaNAC2D was highly homologous to HvSNAC1 (GenBank: AEG21060.1), OsSNAC1(GenBank: AIX03022.1), and almost identical to TaNAC2 (AAU08786.1), indicating that *TaNAC2D*, like these three *NAC* genes (Hu et al., 2006; Mao et al., 2012; Al Abdallat et al., 2014), participated in plant stress responses. The results of Blastn search in *EnsemblPlants* database showed that Traes_5BL_4497A137C.1 had a highest identity (96.8%) of nucleotide sequence with *TaNAC2D*, suggesting that *TaNAC2D* gene was located on chromosome 5BL of wheat.

**FIGURE 1:**
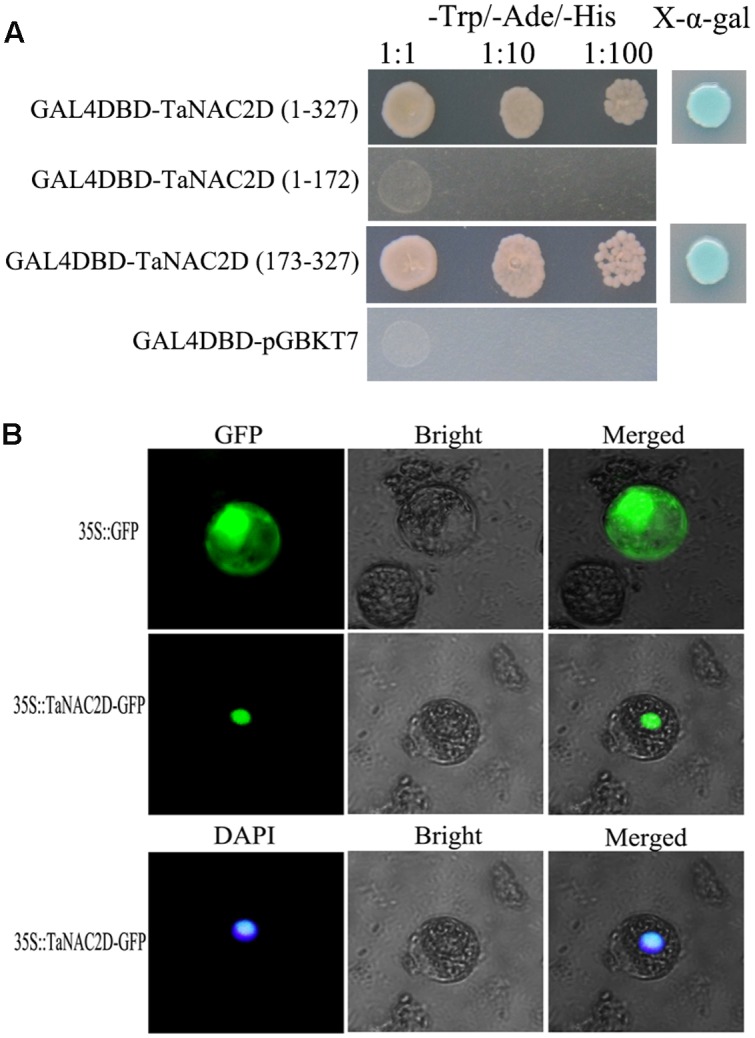
**Transactivation activity and subcellular localization of TaNAC2D.** **(A)** Transactivation activity of TaNAC2D in yeast strain AH109. Full-length and truncated versions of TaNAC2D were fused into pGBKT7 vector and the transformants were screened on the SD/-Trp-Ade-His plates with or without X-α-gal. **(B)** Subcellular localization of TaNAC2D. The fusion protein pMD18-35S-TaNAC2D-GFP and pMD18-35S-GFP (control) were transiently expressed in wheat mesophyll protoplasts and observed with fluorescence microscope.

TaNAC2D contains a NAM domain at N-terminus (amino acids 1∼172) with five subdomains (A–E) and a transcriptional regulatory domain at C-terminus (amino acids 173∼327; **Supplementary Figure S1A**). Transactivation assays demonstrated that TaNAC2D had transactivation activity, and the C-terminus region was enough to activate expression of reporter genes in yeast (**Figure 1A**). TaNAC2D-GFP fusion protein and DAPI were detected only in the nucleus of wheat mesophyll protoplasts (**Figure 1B**). These results indicate that TaNAC2D may function as a transcription factor. Additionally, the result of PONDR VL3 analysis showed that a largely ID region located in C-terminus of TaNAC2D (**Supplementary Figure S1B**), suggesting that the protein was a mostly non-folded conformation at its C-terminus.

### Expression Patterns of *TaNAC2D*

**FIGURE 2:**
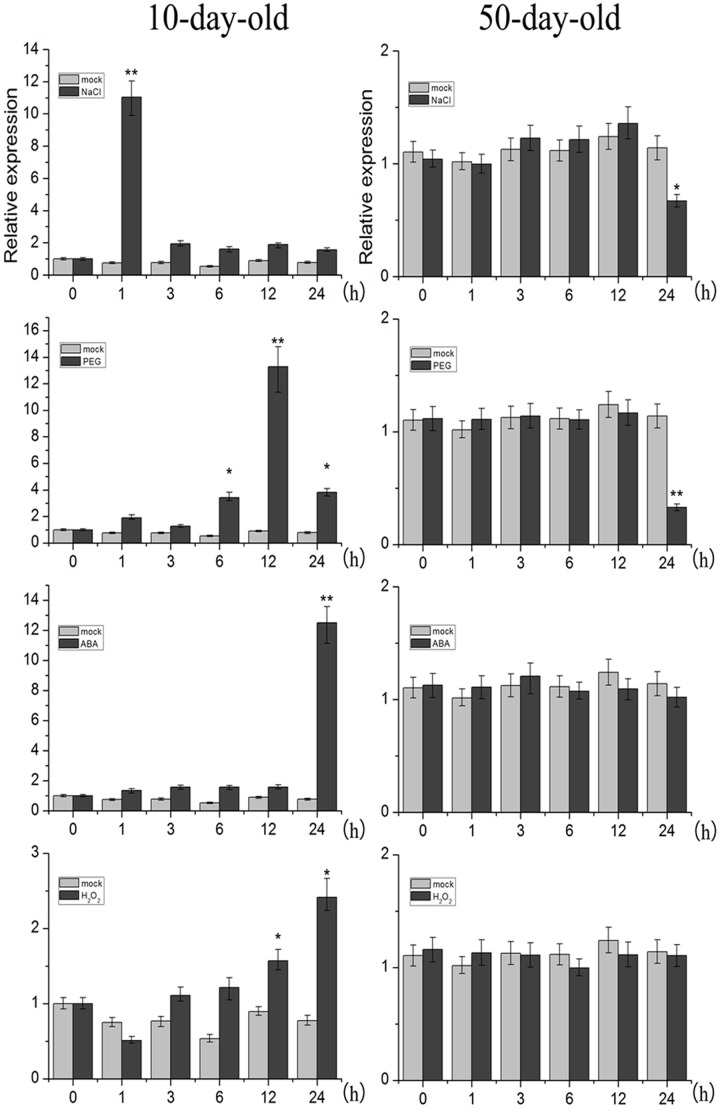
**Expression patterns of *TaNAC2D*.** The 10-day-old wheat seedlings and the 50-day-old mature wheat plants were treated with NaCl, PEG6000, ABA, and H_2_O_2_, transcript levels of *TaNAC2D* in wheat leaves were determined by qRT-PCR and normalized to *TaActin*. Data are means ± SE of three biological replicates. Asterisks indicate significant differences from mock (^∗^*P* < 0.05, ^∗∗^*P* < 0.01).

Organ-specific analysis revealed that *TaNAC2D* transcripts were expressed in all organs except endosperm of wheat, and highly expressed in leaves (**Supplementary Figure S3**). Moreover, we further examined the expression levels of *TaNAC2D* under abiotic stress. The qRT-PCR results showed that *TaNAC2D* was significantly induced by NaCl, PEG, and ABA at seedling stage, and repressed by NaCl and PEG at mature plant stage (**Figure 2**). These results indicate that the expression of *TaNAC2D* is regulated at different stages under stress conditions.

### *TaNAC2D*-OX Plants Exhibit Hypersensitivity to Salt and Drought Stresses at Mature Plant Stage under Non-sterile Conditions

To study the function of gene, *TaNAC2D* was introduced into *Arabidopsis*. Eight transgenic lines (T_3_) were obtained through kanamycin screening. The *TaNAC2D* expression level in T_3_ lines was examined by semi-quantitative PCR analysis. Two *TaNAC2D* high expression lines, designated OX1 and OX8 (**Supplementary Figure S4**), were chosen for further experiments.

**FIGURE 3:**
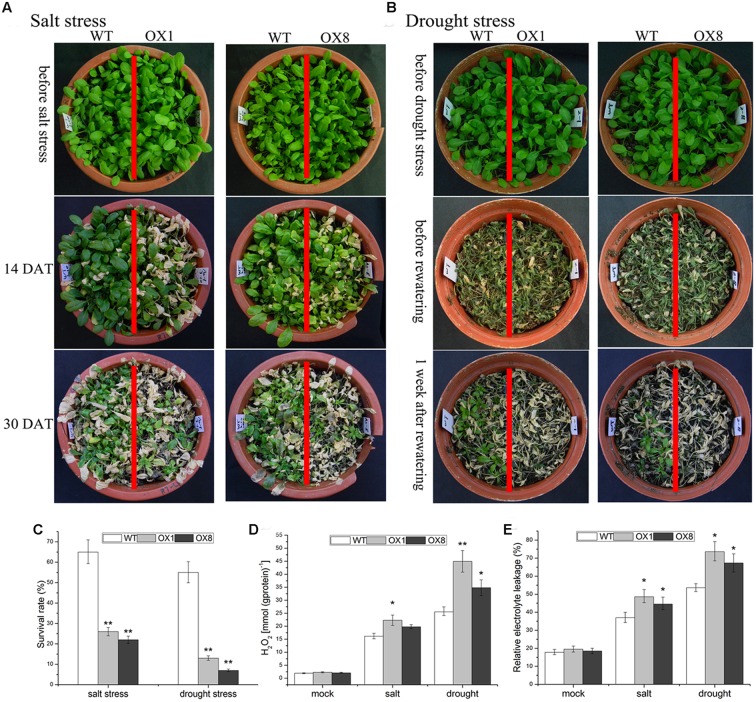
**Hypersensitivity of *TaNAC2D*-OX plants to salt and drought stresses.** **(A)** The 35-day-old WT and *TaNAC2D*-OX plants were irrigated with 200 mM NaCl for 30 days. **(B)** The 35-day-old WT and *TaNAC2D*-OX plants were subjected to drought stress for 20 days and then re-watered for 1 week. **(C)** Survival rate of WT and *TaNAC2D*-OX plants. **(D,E)** Plants were subjected to salt stress for 10 days and drought stress for 17 days, H_2_O_2_ content and relative electrolyte leakage were determined. Data in **(C–E)** are means ± SE of three biological replicates. Asterisks indicate significant differences from control (^∗^*P* < 0.05, ^∗∗^*P* < 0.01).

For the assessment of salt tolerance, when the 35-day-old soil-grown plants were subjected to 200 mM NaCl exposure for 14 days, the leaves of *TaNAC2D*-OX lines began to turn yellow, while the impact of salt stress on WT plants was slightly (**Figure 3A**). After 30 days of salt stress exposure, WT plants had a survival rate of over 60%, whereas OX1 and OX8 showed significantly lower survival rate than WT plants (26 and 22%, respectively; **Figures 3A,C**). This result indicated that *TaNAC2D*-OX plants showed more sensitivity to salt stress than that of WT.

To investigate the impact of drought stress on *TaNAC2D*-OX lines, the 35-day-old soil-grown plants were not irrigated for 20 days, and then re-watered for 7 days. Approximately 90% of *TaNAC2D*-OX lines were dead, whereas over 50% of WT plants still survived (**Figures 3B,C**). This observation indicated that overexpression of *TaNAC2D* resulted in reduced tolerance to drought stress.

To further understand the physiological mechanisms underlying *TaNAC2D* functions that lead to reduced tolerance, H_2_O_2_ content and REL were measured. Under salinity and drought conditions, *TaNAC2D*-OX plants exhibited higher levels of H_2_O_2_ content and REL compared with those of WT plants (**Figures 3D,E**), indicating increased ROS production and greater membrane damage in transgenic plants. These results demonstrated that the *TaNAC2D*-OX plants exhibit hypersensitivity to salt and drought stresses, consistent with the suppression of *TaNAC2D* expression at mature stage of wheat (**Figure 2**).

### *TaNAC2D*-OX Plants Show Reduced Sensitivity to Exogenous ABA

**FIGURE 4:**
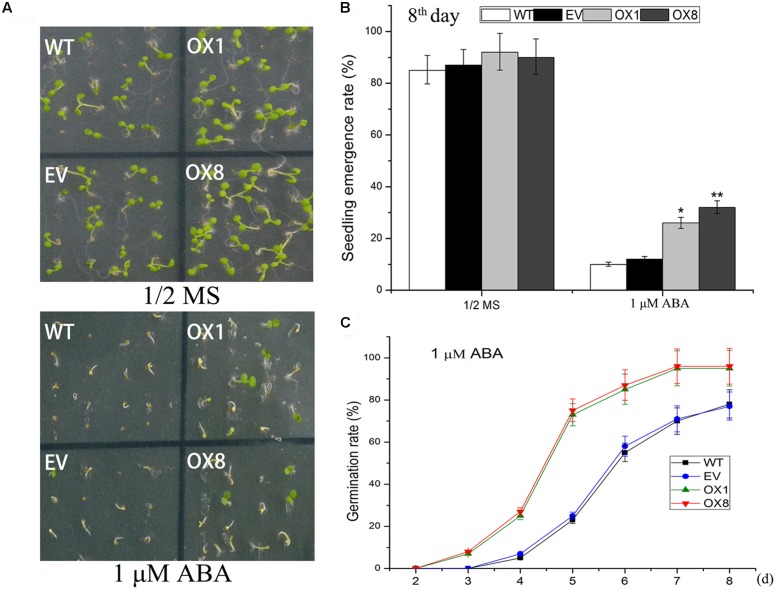
**Response of *TaNAC2D*-OX plants to exogenous ABA.** **(A)** Germination performance of WT, EV, and OXs grown for 8 days on 1/2 MS medium containing 1 μM ABA. **(B)** Seedlings emergence rate of WT, EV, and OXs. **(C)** Percent germination of WT, EV, and OXs. Data in **(B,C)** are means ± SE of three biological replicates (*n* = 40 to 50 seeds per genotype per experiment). Asterisks indicate significant differences from WT (^∗^*P* < 0.05, ^∗∗^*P* < 0.01).

ABA signal is largely regulated by abiotic stress in plants (Yoshida et al., 2014). To investigate if ABA participates in transgenic plants responses to abiotic stresses, we examined responsiveness of *TaNAC2D*-OX plants to exogenous ABA during germination and post-germinative growth periods. The observation showed that exogenous ABA inhibited germination, seedling emergence (seedling with green cotyledon), and root growth in control plants more severely than observed in *TaNAC2D*-OX plants (**Figure 4**; **Supplementary Figure S5**), indicating that *TaNAC2D*-OX plants displayed reduced sensitivity to exogenous ABA than control plants. These results suggested that *TaNAC2D* might be regulated in part by ABA signal during abiotic stress responses in plants.

### Comparison of Water Loss and Stomatal Movement in Control and *TaNAC2D*-OX Plants

**FIGURE 5:**
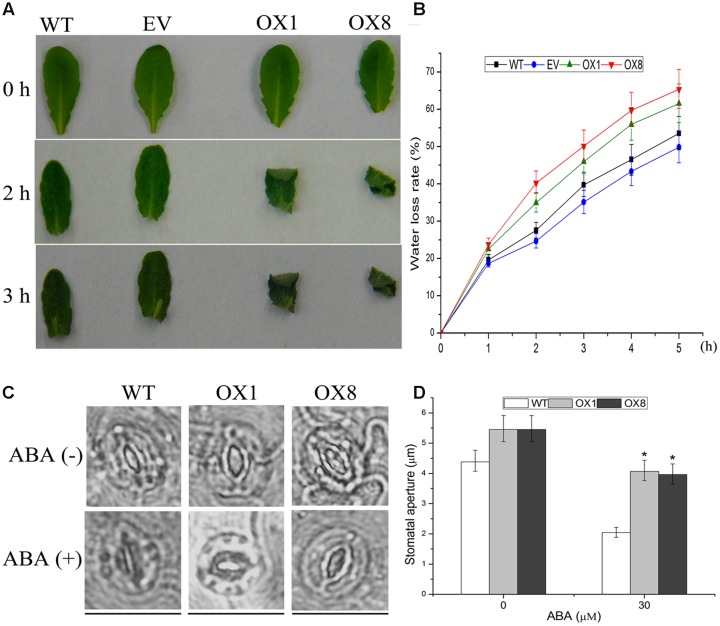
**Response of *TaNAC2D*-OX plants to dehydration and ABA-mediated stomatal closure.** **(A)** Phenotypes of leaves under dehydration stress for 0, 2, and 3 h. **(B)** Water loss rate of WT, EV, and OXs. Data are means ± SE of three independent experiments (*n* = 10). **(C)** Guard cells of 35-day-old soil-grown WT and OXs treated with or without 30 μM ABA for 1 h. Scale bars = 30 μm. **(D)** Quantitative analysis of the size of stomatal aperture. Data are means ± SE (*n* = 50). Asterisks indicate significant differences from WT (^∗^*P* < 0.05).

To further investigate the drought-sensitive phenotype of transgenic lines, detached rosette leaves of the 35-day-old soil-grown plants were sampled for dehydration and ABA-mediated stomatal closure. After 2 or 3 h of air drying, the leaves of OX1 and OX8 were severely curled, whereas the effect of dehydration process on WT and EV plants was slightly (**Figure 5A**). Additionally, *TaNAC2D*-OX lines showed higher water loss rate compared with control plants (**Figure 5B**), indicating that overexpression of *TaNAC2D* in *Arabidopsis* had a decreased water retention capacity.

It is reported that ABA-mediated stomatal movement can affect transpiration rates during dehydration process (Osakabe et al., 2013; Ha et al., 2014). Because *TaNAC2D*-OX plants showed reduced sensitivity to exogenous ABA compared with WT plants (**Figure 4**; **Supplementary Figure S5**), ABA-mediated stomatal movement was further examined. The results revealed that the stomata of *TaNAC2D*-OX plants closed more slowly compared with that of WT plants (**Figures 5C,D**), indicating that the slower stomatal closure observed in *TaNAC2D*-OX plants was directly responsible for greater water loss and increased drought sensitivity of transgenic plants.

### *TaNAC2D*-OX Plants Show Increased Tolerance to Salt, Osmotic and Oxidative Stresses at Seedling Stage under Sterile Conditions

**FIGURE 6:**
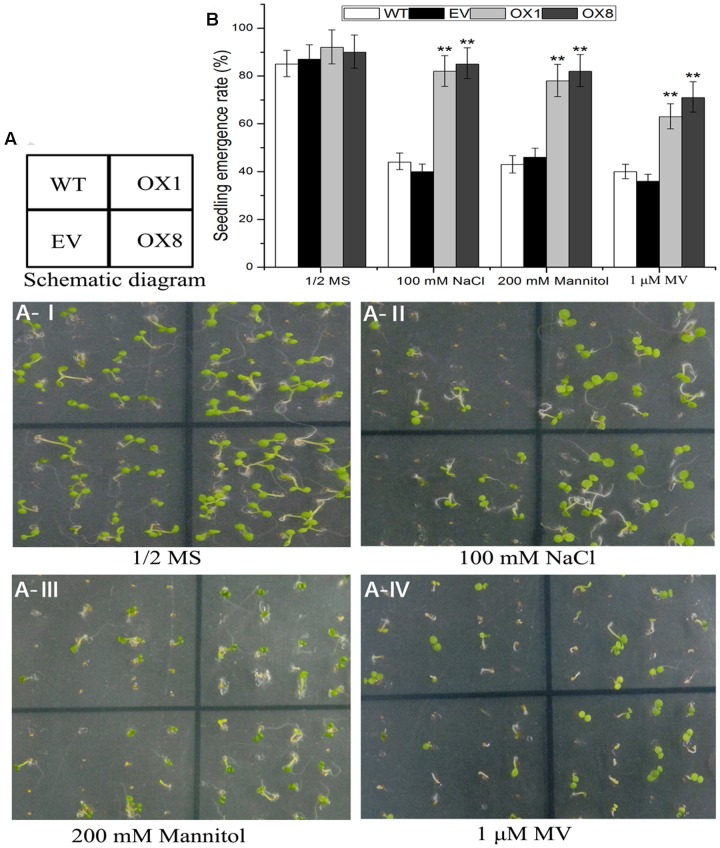
**Seedling emergence assays of WT, EV and *TaNAC2D*-OX plants.** **(A)** Seedling emergence performance of WT, EV, and OXs grown for 8 days on 1/2 MS medium (control) or 1/2 MS medium containing 100 mM NaCl, 200 mM Mannitol, and 1 μM MV. **(B)** Seedlings emergence rate of WT, EV, and OXs. Data are means ± SE of three biological replicates (*n* = 40 to 50 seeds per genotype per experiment). Asterisks indicate significant differences from WT (^∗∗^*P* < 0.01).

Several studies reveal that stress tolerance of plants is associated with germination and root growth of seedlings (Ren et al., 2010; Zhu et al., 2010). To further investigate mechanisms of hypersensitivity to abiotic stress in *TaNAC2D*-OX plants, we examined germination, primary root length, and plant survive in control and *TaNAC2D*-OX lines under salt, osmotic and oxidative stresses. The statistical analysis of seed germination showed that *TaNAC2D*-OX lines grown on 1/2 MS medium supplemented with 100 mM NaCl, 200 mM mannitol or 1 μM MV, had higher germination rate than control plants (**Figure 6**). Root growth also exhibited that *TaNAC2D*-OX lines had longer primary root compared with control plants under salt and osmotic stresses (**Supplementary Figure S6**). Moreover, root growth in the control and *TaNAC2D*-OX lines was inhibited to similar extents by MV treatment, whereas *TaNAC2D*-OX lines had a higher survival rate than control plants under oxidative stress (**Supplementary Figure S6**). These results suggested that *TaNAC2D*-OX plants had improved tolerance to salt, osmotic and oxidative stresses during seed germination and post-germinative growth periods, consistent with the induced expression of *TaNAC2D* at seedling stage of wheat (**Figure 2**).

### Expression Analysis of *NCED3*, *RD29A*, and *RD29B* in WT and *TaNAC2D*-OX Plants under Salt and Dehydration Conditions

**FIGURE 7:**
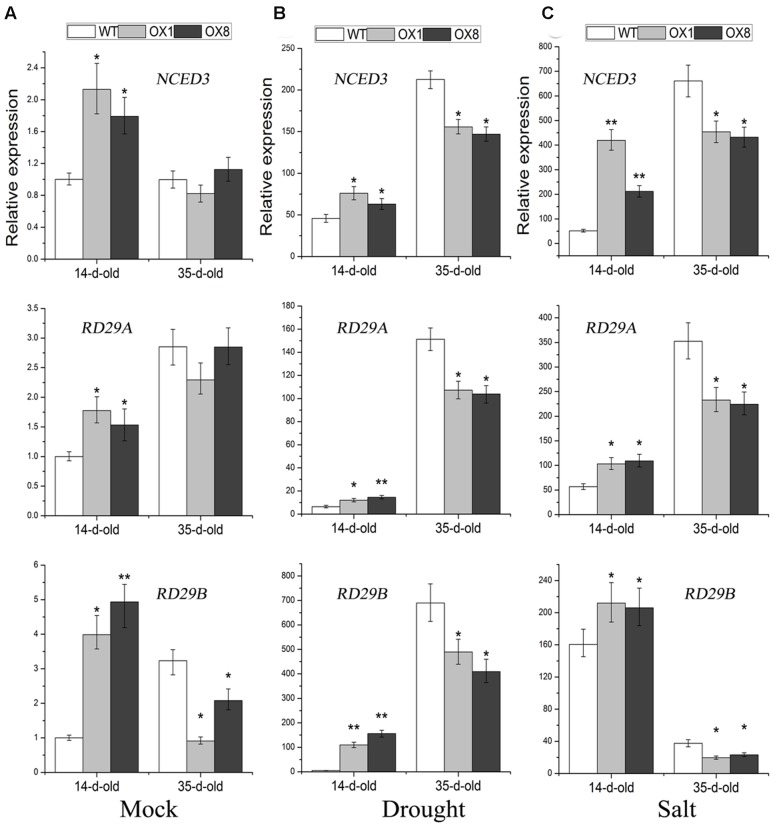
**Expression pattern of *NCED3*, *RD29A* and *RD29B* in WT and *TaNAC2D*-OX plants.** The 14-day-old seedlings grown on 1/2 MS medium were subsequently submerged in 1/2 MS liquid cultures supplemented with 200 mM NaCl for 6 h or placed on dry filter paper, and then dehydrated for 1 h. Moreover, the 35-day-old soil-grown plants were incubated for 6 h in 200 mM NaCl solution or dehydrated for 3 h. Transcript levels were determined by qRT-PCR and normalized to *actin2*. The values in 14-day-old untreated WT plants were set to 1. Data are means ± SE of three biological replicates. Asterisks indicate significant differences from WT (^∗^*P* < 0.05; ^∗∗^*P* < 0.01). **(A)** Effects of normal growth. **(B)** Effects of drought stress. **(C)** Effects of salt stress.

Many stress-inducible marker genes that are up-regulated in plants are expected to increase plant tolerance to abiotic stress (Cramer et al., 2011; Yoshida et al., 2014). To further understand the above-mentioned different stress-responsive phenotypes of *TaNAC2D*-OX plants, the expression analysis of marker genes, including *NCED3*, *RD29A*, and *RD29B*, was further performed. The 14-day-old seedlings, grown on 1/2 MS medium, were untreated (control) or treated with NaCl (200 mM) for 6 h or dehydration for 1 h, respectively, the transcript levels of *NCED3*, *RD29A* and *RD29B* were all significantly up-regulated in *TaNAC2D*-OX plants than in WT plants (**Figures 7A–C**); this was consistent with the stress-tolerant phenotypes of *TaNAC2D*-OX plants during germination and post-germinative growth periods (**Figure 6**; **Supplementary Figure S6**). In contrast, the 35-day-old soil-grown plants were incubated for 6 h in salt solution or dehydrated for 3 h, respectively, the transcript levels of *NCED3*, *RD29A* and *RD29B* were all significantly down-regulated in *TaNAC2D*-OX plants compared with in WT plants (**Figures 7B,C**); this was also in accordance with the stress-sensitive phenotypes of soil-grown *TaNAC2D*-OX plants (**Figures 3A,B**). These results suggested that the different growth stages and environmental conditions may regulate the transcriptional activity of TaNAC2D, leading to up-regulating or down-regulating of downstream genes, such as *NCED3*, *RD29A*, and *RD29B*.

## Discussion

### TaNAC2D Is a NAC TFs that Participates in the Abiotic Stress Response

Many NAC TFs have been shown to participate in plant stress responses (Nakashima et al., 2012). In *Arabidopsis*, plant overexpressing *ATAF1*, *ANAC019*, *ANAC055*, or *ANAC072* confers an improved ability to drought stress (Tran et al., 2004; Wu et al., 2009). Overexpression of *VNI2*/*ANAC083* and *JUB1*/*ANAC042* has enhanced tolerance to abiotic stress, meanwhile these two genes are also involved in senescence process (Yang et al., 2011; Wu et al., 2012). In rice (*O. sativa*), plant overexpressing either *SNAC1*, *OsNAC5*, *OsNAC9*, or *OsNAC10* increases stress tolerance of transgenic rice (Hu et al., 2006; Jeong et al., 2010, 2013; Redillas et al., 2012). In wheat, overexpression of *TaNAC2*, *TaNAC29*, *TaNAC67*, and *TaNAC69* exhibits stress-tolerant phenotypes of transgenic plants (Xue et al., 2011; Mao et al., 2012, 2014; Huang et al., 2015).

In this study, phylogenetic analysis of TaNAC2D showed a close relationship with TaNAC2, HvSNAC1 and OsSNAC1. The analysis of NCBI BLASTP revealed that TaNAC2D had 98% identity to TaNAC2 from *T. aestivum*, 95% identity to HvSNAC1 from *Hordeum vulgare*, 71% identity to OsSNAC1 from *O. sativa*. Transgenic *Arabidopsis* plants overexpressing *TaNAC2* increase tolerance to salt, drought and cold stresses compared with WT plants (Mao et al., 2012). The present study showed that *Arabidopsis* plants overexpressing *TaNAC2D* displayed increased sensitivity to salt and drought stresses at mature stage, and only improved stress tolerance at seedling stage. Our results are only partly in agreement with the previous findings of TaNAC2. In addition, overexpression of *HvSNAC1* in barley results in an increased resistance to drought and *Ramularia* leaf spot in the transgenic plants (Al Abdallat et al., 2014; McGrann et al., 2015). The transgenic barley plants overexpressing *HvSNAC1* also delay dark-induced leaf senescence (McGrann et al., 2015). OsSNAC1 is the rice ortholog of TaNAC2D. Overexpression of *OsSNAC1* in rice improves tolerance to drought and salt stresses at the vegetative stage (Hu et al., 2006). These previous findings together with our data imply that *TaNAC2D* may be involved in the stress response, pathogen defense, and senescence. In the present study, ectopic expression of *TaNAC2D* in *Arabidopsis* is a faster attempt to detect gene functions. However, the real role of gene needs to be further validated by overexpression and knock-down of *TaNAC2D* in wheat. We presume that *TaNAC2D*, like *HvSNAC1* and *OsSNAC1* (Hu et al., 2006; Al Abdallat et al., 2014; McGrann et al., 2015), is also involved in biotic and abiotic stress response in wheat.

### Overexpression of *TaNAC2D* in *Arabidopsis* Reduces Drought Stress Tolerance at Mature Plant Stage

In the present study, leaves of 35-day-old soil-grown *TaNAC2D*-OX plants were found to be curled more severely and to lose water more rapidly compared with leaves of control plants when they were treated with dehydration, indicating that *TaNAC2D*-OX plants exhibited reduced drought tolerance than control plants due to the higher transpiration rate. The higher transpiration rate in *TaNAC2D*-OX plants was significantly correlation with the slower stomatal closure during dehydration. Additionally, *TaNAC2D*-OX plants displayed hyposensitivity to exogenous ABA during the germination and post-germinative growth periods. The slower ABA-mediated stomatal closure might be caused by ABA hyposensitivity, rendering more water loss and the increased drought sensitivity of *TaNAC2D*-OX plants. Several studies have reported that drought tolerance is associated with the transpiration rate of leaf and degree of stomatal closure. For example, *max* (*more axillary growth*) mutant plants exhibit hyposensitivity to exogenous ABA, the slower ABA-mediated stomatal closure, the higher transpiration rate, and the reduced drought tolerance compared with WT plants (Ha et al., 2014). *KUP6*, *KUP8* and *GORK* mutant plants display decreased sensitivity to exogenous ABA, impaired stomatal closure, and reduced tolerance to drought stress (Osakabe et al., 2013). Consistent with the findings of previous studies, overexpression of *TaNAC2D* in *Arabidopsis* also exhibited the same characteristic.

### Changes in Growth Stages and Environmental Conditions Alter Stress-Responsive Phenotypes of *TaNAC2D*-OX Plants

Phylogenetic analysis showed that TaNAC2D had close homology to ATAF1 and ATAF2 from *Arabidopsis* (**Supplementary Figure S7**), suggesting that TaNAC2D might have the similar function with them. Several studies have demonstrated the biological functions of ATAF1 and ATAF2. In one study, *ATAF1*-OX plants exhibit enhanced drought tolerance, and increased sensitivity to high salinity and oxidative stresses (Wu et al., 2009). Contrastingly, the earlier studies reveal that *ataf1* mutant plants show improved drought tolerance (Lu et al., 2007; Jensen et al., 2008). Recently, two new studies show that ATAF1 directly interacts with the promoter of *NCED3*, which encodes a key enzyme in ABA biosynthesis pathway (Jensen et al., 2013; Garapati et al., 2015). Overexpression of *ATAF1* improves the transcript level of *NCED3*, leading to an increased endogenous ABA level, while *ataf1* mutant plants exhibit a significantly decreased *NCED3* expression compared with WT plants (Jensen et al., 2013). The increased endogenous ABA level contributes to the enhanced tolerance of plants against abiotic stress (Jensen et al., 2013). Thus, the increased *NCED3* expression level is responsible for the enhanced drought tolerance of *ATAF1*-OX plants observed by Wu et al. (2009), but cannot explain an increased drought tolerance of *ataf1* mutants observed by Lu et al. (2007) and Jensen et al. (2008). These previous studies suggest that TaNAC2D may also be involved in regulating *NCED3* expression because TaNAC2D had a close homology to ATAF1. Moreover, two abiotic stress-inducible marker genes, including *RD29A* and *RD29B*, are involved in ABA signaling pathway (Yamaguchi-Shinozaki and Shinozaki, 1993; Mizoguchi et al., 2010). *ANAC083*-OX seedlings displays enhanced salt tolerance, meanwhile the transcript levels of *RD29A* and *RD29B* are up-regulated in *ANAC083*-OX seedlings under salt stress (Yang et al., 2011). Thus, the comparative analysis of *NCED3*, *RD29A* and *RD29B* expression levels in WT and *TaNAC2D*-OX plants was conducted to detect the molecular mechanisms of *TaNAC2D* function. The results suggest that the down-regulation of *NCED3*, *RD29A*, and *RD29B* may be responsible for the reduced stress tolerance observed in the 35-day-old soil-grown *TaNAC2D*-OX plants. In contrast, the up-regulation of these three marker genes in 14-day-old *TaNAC2D*-OX seedling, grown on 1/2 MS medium, may contribute to the enhanced stress tolerance during germination and post-germinative growth periods. Our results indicate that the changes in growth stages and environmental conditions affect the expression levels of *NCED3*, *RD29A* and *RD29B* by regulating the transcriptional activity of TaNAC2D, leading to the variant stress-responsive phenotypes of *TaNAC2D*-OX plants. Several studies have demonstrated that the different growth stages and environmental conditions may regulate gene functions. For example, ATAF2 functions as a negative regulator of pathogenesis-related genes under sterile conditions (Delessert et al., 2005). In contrast, ATAF2 can increase the expression of pathogenesis-related genes under non-sterile growth conditions (Wang et al., 2009). Ha et al. (2014) revealed that the *max* mutant plants display increased sensitivity to salt and drought stresses compared with WT plants at mature plant stage, while primary root length is not significantly different between WT and *max* mutant plants at seedling stage under salt and osmotic stresses conditions. Based on previous findings and our own data, TaNAC2D’s function in the regulation of the stress response may vary depending on the growth stages and environmental conditions of plant.

Several NAC TFs act either as transcriptional repressors or activators to regulate the expression of downstream genes, leading to down-regulation or up-regulation of these target genes. VNI2 is a transcriptional repressor under normal growth conditions (Yamaguchi et al., 2010). However, the transcriptional activation activity of VNI2 was greatly elevated under salt-treatment conditions, resulting in up-regulation of *COR* and *RD* genes (Yang et al., 2011). *Arabidopsis* WUSCHEL acts as a repressor of transcription in shoot meristems, but it becomes an activator of transcription in floral patterning (Leibfried et al., 2005; Ikeda et al., 2009). These previous findings reveal that the transcriptional regulation activities are modulated by both intrinsic and environmental factors. Moreover, NAC TFs contain DNA-binding (DB) domain, NAC repression domain (NARD) and activation domain, which interact with each other to regulate transcriptional activities (Hao et al., 2010; Puranik et al., 2012). The D subdomain of NAC (**Supplementary Figure S1A**) contains NARD which acts to suppress transcriptional activity (Hao et al., 2010). If the function of NARD is stronger than that of activation domain, NAC TFs may be a transcriptional repressor. Conversely, NAC TFs act as a transcriptional activator. If the functions from NARD and activation domain are roughly equal, the transcriptional regulation activities may be dependent on the actual situation in plant (Hao et al., 2010). It is possible that the growth stages and environmental conditions may induce changes in the structure and/or activities of TaNAC2D. Based on previous findings and our data, we propose that TaNAC2D acts as a transcriptional activator at seedling stage under sterile conditions and as a transcriptional repressor at mature plant stage under non-sterile conditions.

## Conclusion

Our results show that TaNAC2D encodes a typical NAC TFs. The 35-day-old soil-grown *TaNAC2D*-OX plants displayed greater water loss and significantly reduced salt and drought tolerance under non-sterile conditions, while *TaNAC2D*-OX seedlings had improved tolerance to salt, drought, and oxidative stresses at seedling stage under sterile conditions. The different growth stages and environmental conditions may induce change in the transcriptional activity of TaNAC2D, leading to up-regulation or down-regulation of downstream genes. The present study provides some insight into the complex mechanisms of NAC.

## Author Contributions

QH conducted all experiments, analyzed the data, and drafted the manuscript. YW helped to conduct experiments. All authors read and approved the final manuscript.

## Conflict of Interest Statement

The authors declare that the research was conducted in the absence of any commercial or financial relationships that could be construed as a potential conflict of interest.

## Footnotes

1 http://www.ebi.ac.uk/interpro/search/sequence-search

2 http://www.pondr.com

## Abbreviations

ABA: abscisic acid
EV: empty vector
ID: intrinsically disordered
MV: methylviologen
NAC: NAM, ATAF and CUC
ORF: open reading frame
OX: overexpression
qRT-PCR: quantitative reverse transcription-PCR
REL: relative electrolyte leakage
WT: wild-type
