# Supplementary material for: Overexpression of TaNAC2D Displays Opposite Responses to Abiotic Stresses between Seedling and Mature Stage of Transgenic Arabidopsis
Source: Front Plant Sci. 2016 Nov 23;7:1754. doi: 10.3389/fpls.2016.01754 (PMC5120104; doi:10.3389/fpls.2016.01754)
Supplement: Supplementary file 1 [file Presentation_1.PDF]

**Supplementary Table S1. Primers used in the present study**

(A) Primers used for cloning cDNA of *TaNAC2D* from wheat

| Gene Name | Forward/reverse primers |
|---|---|
| *TaNAC2D* | 5'-CCACAATCTCAACAGCCATCGGA-3' |
| | 5'-CCCTCCCGTTTCCTATGCTGTA-3' |

(B) Primers used for qRT-PCR analysis of *TaNAC2D* gene expression

| Gene Name | Forward/reverse primers |
|---|---|
| *TaNAC2D* | 5'-ACCTCAGCTACGACGACATCCAG-3' |
| | 5'-GCGGCGAAGAAGTCATCCGTTCC-3' |
| *TaActin* | 5'-TCTATTTTGGCCTCTCTTAGCAC-3' |
| | 5'-TTTCCTGTACCCCTTATTCCTC-3' |

(C) Primers used in subcellular localization assay of TaNAC2D

| Gene Name | Forward/reverse primers | Restriction enzymes |
|---|---|---|
| *TaNAC2D* | 5'-TGCTCTAGAATGGGGATGCCGGCGGTGAGG-3' | *Xba*I |
| | 5'-TCCCCCGGGGAACGGGGCCGGCAT-3' | *Sma*I |

(D) Primers used in transactivation activity analysis of TaNAC2D

| GAL4DBD–NAC | Forward/reverse primers | Restriction enzymes |
|---|---|---|
| *TaNAC2D*$_{1\text{-}327}$ | 5'-CCGGAATTCATGGGGATGCCGGCGGTGAGG-3' | *Eco*R1 |
| | 5'-CGCGGATCCTCTGTGTCTTGGCTCTGCTTA-3' | *Bam*H1 |
| *TaNAC2D*$_{1\text{-}172}$ | 5'-CCGGAATTCATGGGGATGCCGGCGGTGAGG-3' | *Eco*R1 |
| | 5'-CGCGGATCCCTTGTTGTAGAGCCGGCAGAG-3' | *Bam*H1 |
| *TaNAC2D*$_{173\text{-}327}$ | 5'-CCGGAATTCAAGAACGAGTGGGAGAAGATG-3' | *Eco*R1 |
| | 5'-CGCGGATCCTCTGTGTCTTGGCTCTGCTTA-3' | *Bam*H1 |

(E) Primers used for cloning *TaNAC2D* into pBI121 vector, and for semi-quantitative RT-PCR analysis

| | Forward/reverse primers | Restriction enzymes |
|---|---|---|
| *pBI121-TaNAC2D* | 5'-TGCTCTAGAATGGGGATGCCGGCGGTGAGG-3' | *Xba*I |
| | 5'-TCCCCCGGGTTAGAACGGGGCCGGCAT-3' | *Sma*I |
| semi-quantitative | 5'-ACCTCAGCTACGACGACATCCAG-3' | |
| RT-PCR analysis | 5'-TTCGCGCTGATACCAGACGTT-3' | |
| *Actin2* | 5'-GGTAACATTGTGCTCAGTGGTGG-3' | |
| | 5'-AACGACCTTAATCTTCATGCTGC-3' | |

(F) Primers used in qRT-PCR for gene expression analyses of marker genes

| Gene name | Forward/reverse primers |
|---|---|
| *actin2* | 5'-GGTAACATTGTGCTCAGTGGTGG-3' |
| | 5'-AACGACCTTAATCTTCATGCTGC-3' |
| *RD29A* | 5'-GTTACTGATCCCACCAAAGAAGA-3' |
| | 5'-GGAGACTCATCAGTCACTTCCA-3' |
| *RD29B* | 5'-GGAGTTCAAGATTCTGGGAAC-3' |
| | 5'-CATCAAAGTTCACAAACAGAGGC-3' |
| *NCED3* | 5'-AGTCAACCTCGAAGCAGGGATG-3' |
| | 5'-CTCGGCTAAAGCCAAGTAAGCG-3' |

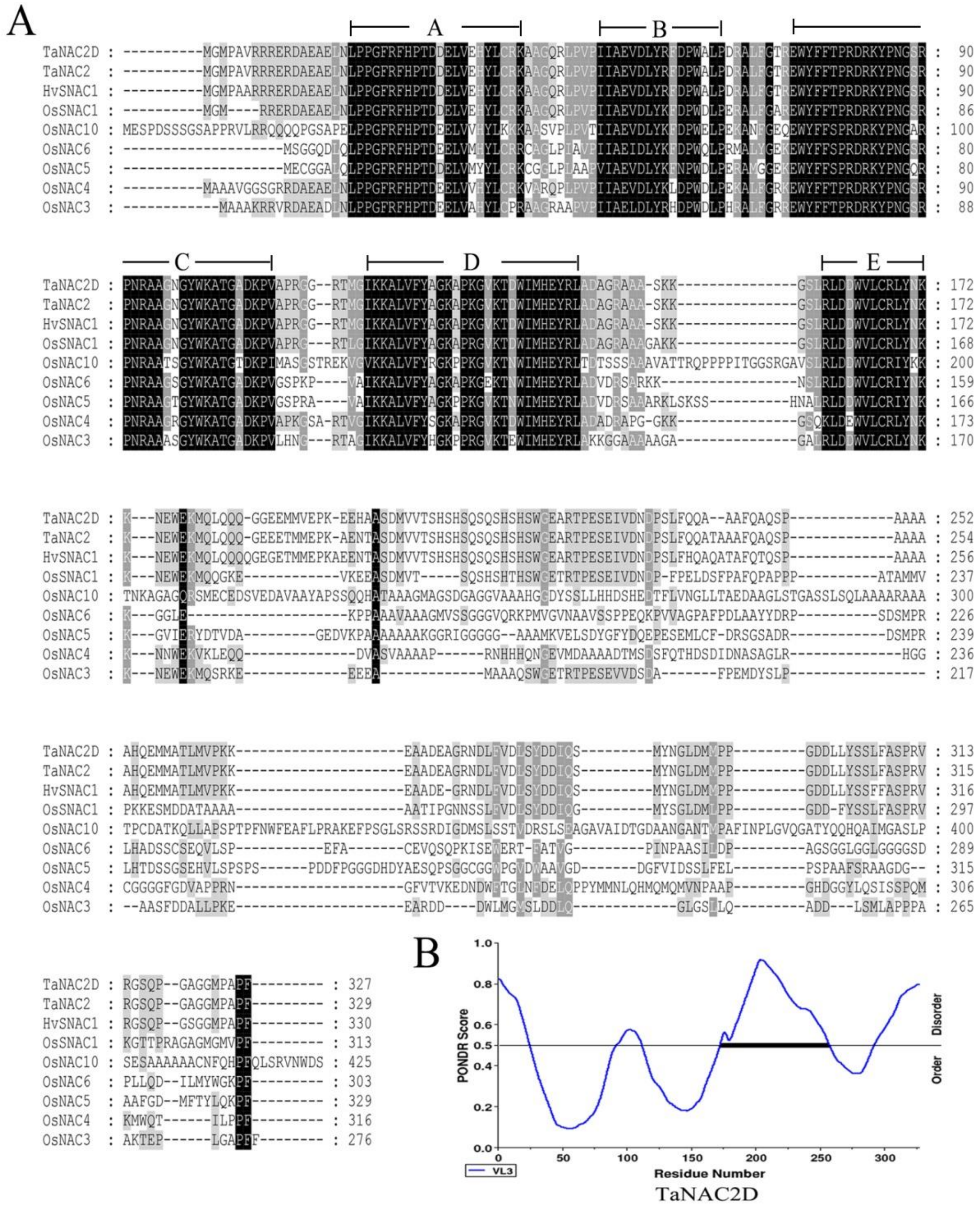


**Fig. S1. Structural features of TaNAC2D**

(A) Amino acid sequences alignment of TaNAC2D and other related NAC members from selected plant species. Five conserved NAC-subdomain were indicated by capital letters. Alignments were performed using MEGA5.1 program. (B) PONDR VL3 analysis of TaNAC2D. An intrinsically disordered region of TaNAC2D were indicated by the black bar.

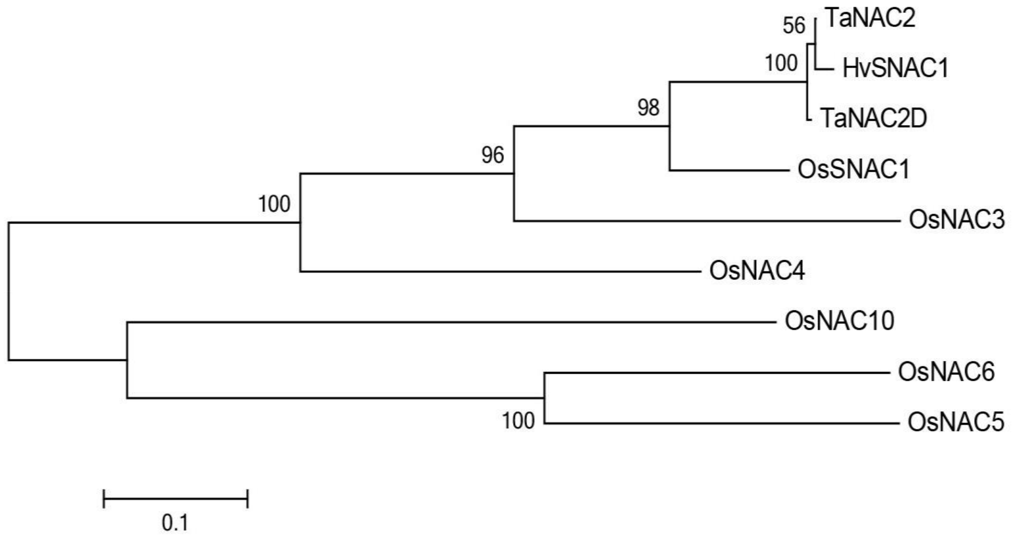


**Fig. S2. Phylogenetic analysis of TaNAC2D with other NAC members** Phylogenetic analysis of TaNAC2D (GenBank: ADE59447.1) with TaNAC2 (GenBank: AAU08786.1) from *Triticum aestivum*, HvSNAC1 (GenBank: AEG21060.1) from *Hordeum vulgare*, OsSNAC1(GenBank: AIX03022.1), OsNAC10 (GenBank: A2YMR0.1), OsNAC6 (GenBank: BAA89800.1), OsNAC5 (GenBank: BAA89799.1), OsNAC3 (GenBank: BAA89797.1) and OsNAC4 (GenBank: BAA89798.1) from *Oryza sativa*.

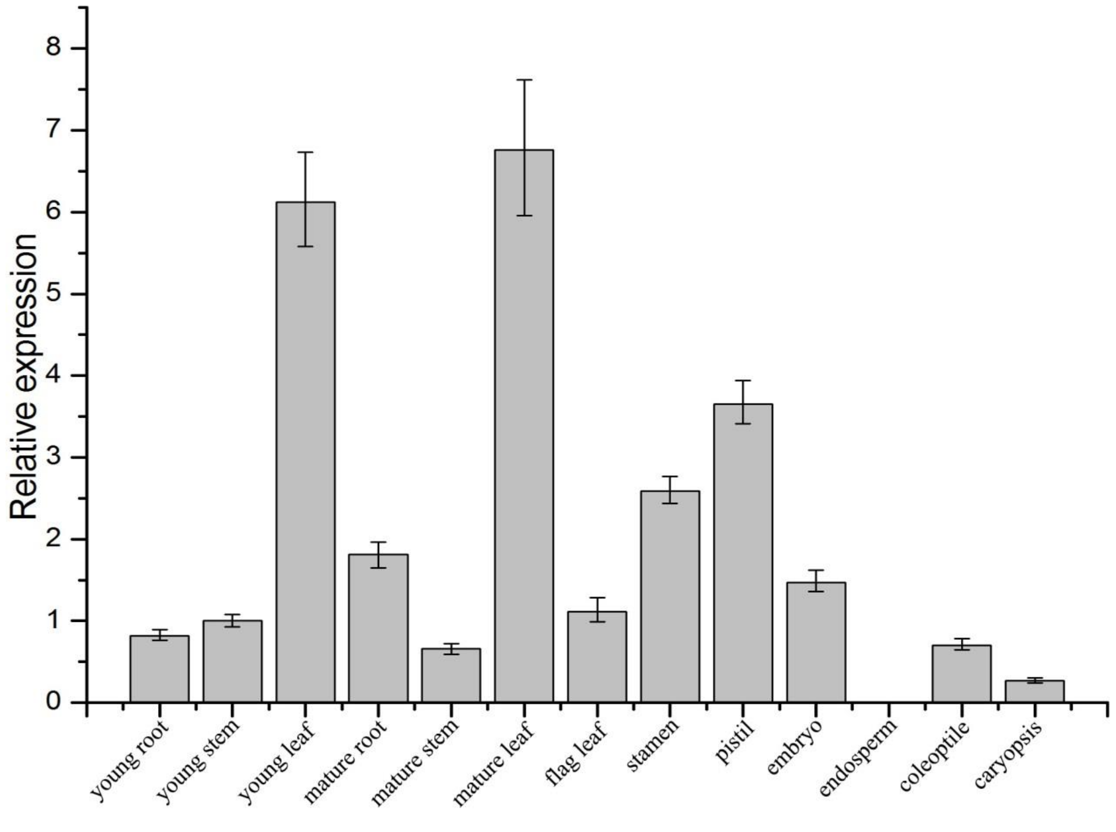


**Fig. S3. qRT-PCR analysis of *TaNAC2D* expression in wheat organ**

Different organs include root, stem, leaf (at four-leaf stage and anthesis stage); flag leaf; stamen; pistil; embryo; endosperm; coleoptile; caryopsis. Data are means ±SE of three replicates.

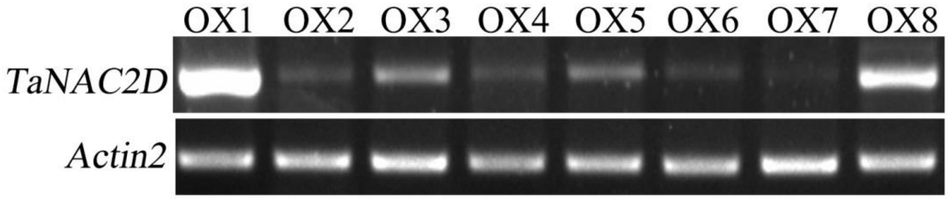


**Fig. S4. Expression of *TaNAC2D* in transgenic *Arabidopsis* lines**

Total RNAs were extracted from 14-d-old transgenic *Arabidopsis* lines grown on 1/2 MS medium. *Actin2* was included as the internal control for examining the gene overexpression (OX) of *TaNAC2D*. Three independent experiments were performed.

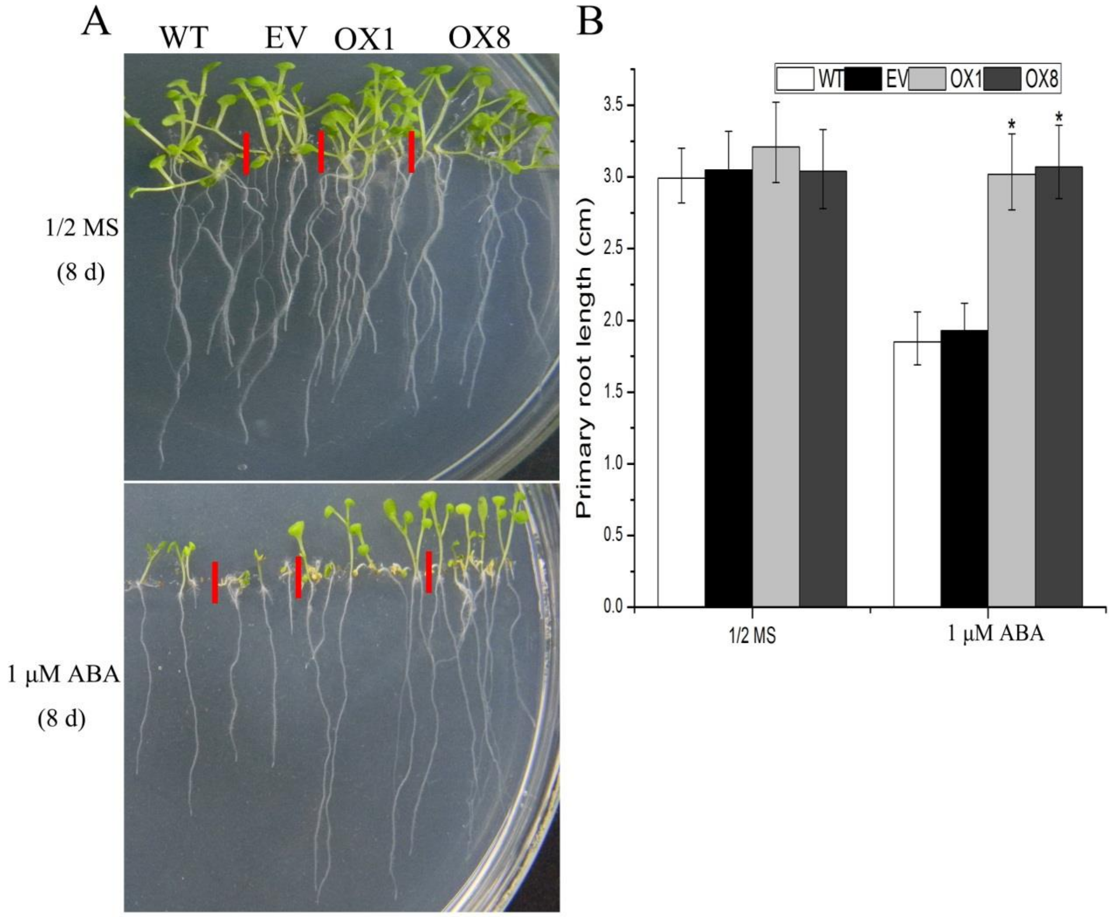


**Fig. S5. Effect of ABA on root growth of WT, EV and *TaNAC2D*-OX plants**

(A) Phenotypes of WT, EV and OXs grown for 8 d on 1/2 MS medium containing 0 or 1 μM ABA. (B) Quantitative analysis of primary root length. Data are means ±SE of three biological replicates ($n$ = 20 to 25 plants per genotype per experiment). Asterisks indicate significant differences from WT (*$P<0.05$).

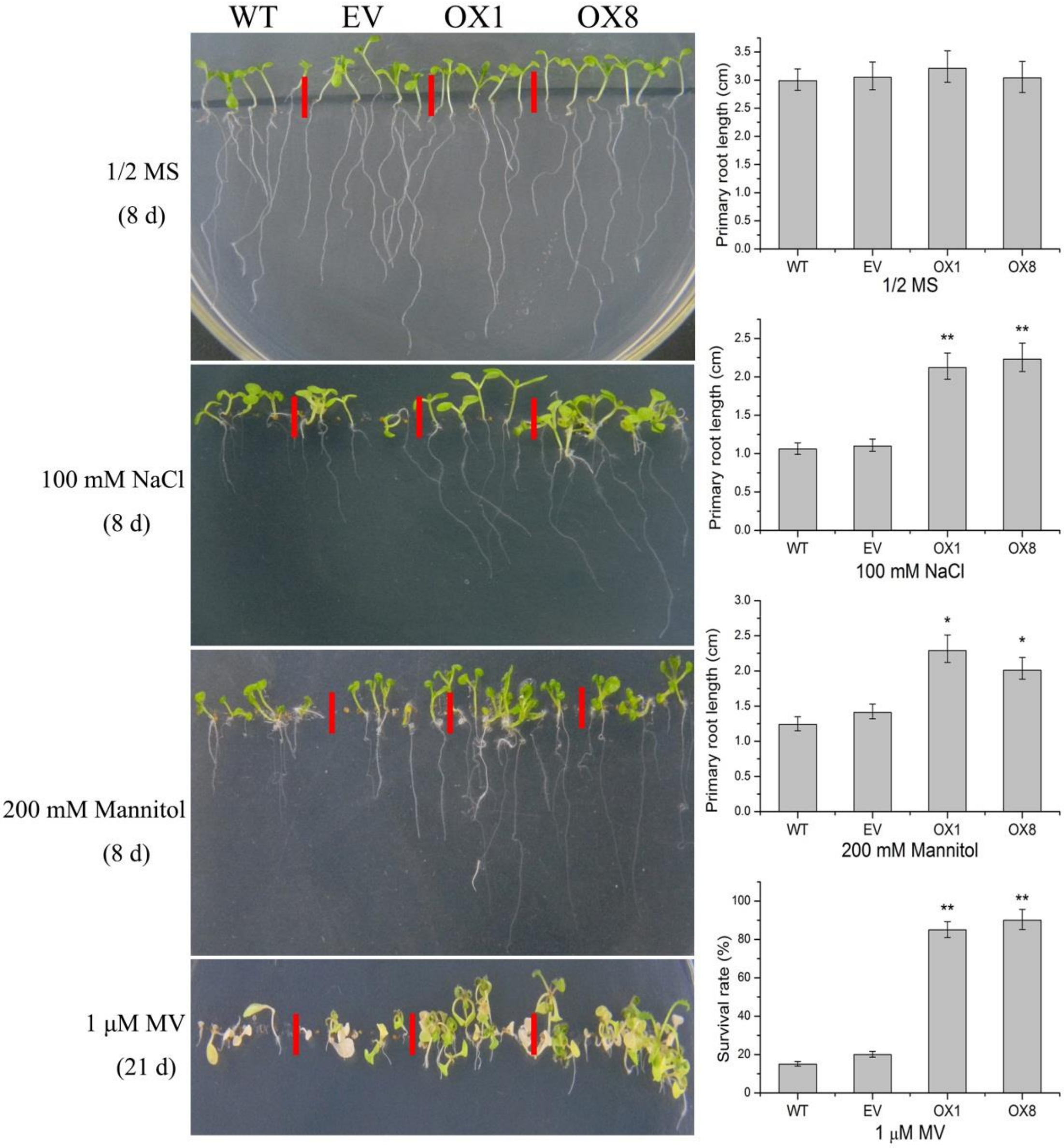


**Fig. S6. Root growth or plant survival of WT, EV and *TaNAC2D*-OX plants**

Left, phenotypes of WT, EV, and OXs grown for 8 d on 1/2 MS medium (control) or 1/2 MS medium containing 100mM NaCl, 200mM Mannitol, and 1 μM MV. Right, primary root length of WT, EV and OXs treated with NaCl and Mannitol, and survival rate analysis of MV treatment. Data are means ±SE of three biological replicates (*n* = 20 to 25 plants per genotype per experiment). Asterisks indicate significant differences from WT (**P*<0.05; ***P*<0.01).

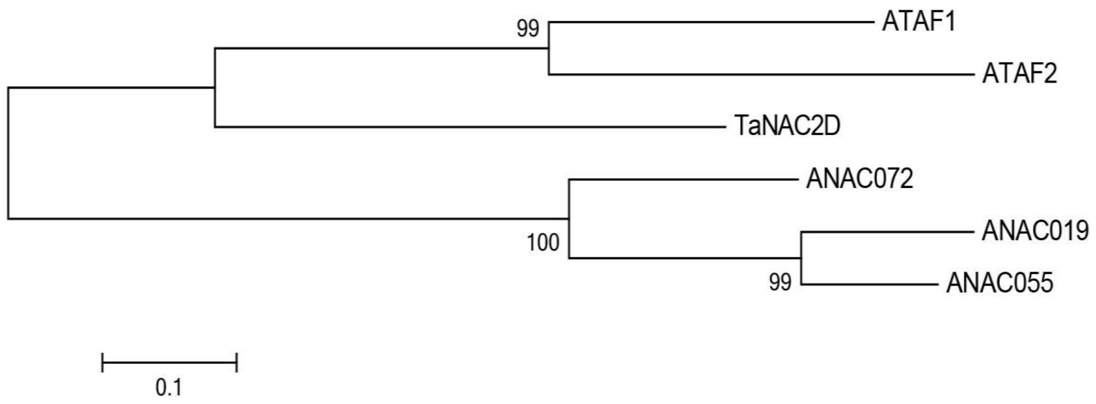


**Fig. S7. Phylogenetic analysis of TaNAC2D with other NAC members from *Arabidopsis thaliana***

The accession numbers of NAC proteins are as follows: TaNAC2D (GenBank: ADE59447.1), ATAF1 (AT1G01720), ATAF2 (AT5G08790), ANAC019 (AT1G52890), ANAC055 (AT3G15500), ANAC072 (AT4G27410).
